# Efficacy of Topical Tacrolimus (FK506) in High-risk Penetrating Keratoplasty: A Systematic Review and Meta-analysis of Comparative Studies

**DOI:** 10.1038/s41433-025-04002-x

**Published:** 2025-10-26

**Authors:** Jumanah Qedair, Razan Bawazir, Saeed Alghamdi

**Affiliations:** 1 College of Medicine, King Saud bin Abdulaziz University for Health Sciences, Jeddah, Saudi Arabia; 2 King Abdullah International Medical Research Center (KAIMRC), Jeddah, Saudi Arabia; 3 Department of Ophthalmology, King Faisal Specialist Hospital and Research Center, Jeddah, Saudi Arabia

**Keywords:** Corneal diseases, Vision disorders

## Abstract

Corneal transplant rejection poses a significant challenge for patients undergoing high-risk penetrating keratoplasty (HR-PKP). We systematically reviewed the literature to evaluate the efficacy of combining topical tacrolimus with steroids in reducing graft failure rates. A systematic review and meta-analysis were conducted according to PRISMA guidelines. PubMed, Web of Science and CENTRAL/Cochrane databases were searched from inception to September 2024. Meta-analyses were conducted using weighted means, proportions and log odds ratios (OR) with a random-effects model. A two-tailed p-value of <0.05 was considered statistically significant. Out of 653 articles identified initially, five homogeneous comparative studies (n = 274 HR-PKP patients) were included. The tacrolimus-plus-steroids (n = 138) and steroids-alone (n = 136) groups were well-matched in baseline characteristics: weighted mean age (54.0 vs. 52.6 years, p = 0.90), sex distribution (p = 0.68), and underlying diagnoses with keratoconus being the most common (29.7% vs. 25.6%, p = 0.68). Follow-up durations were comparable as well (20.1 vs. 20.2 months, p = 0.98). After undergoing HR-PKP, patients in the tacrolimus-plus-steroids group showed significantly reduced graft failure odds by 75.0% (OR: 0.25 [95% CI: 0.13–0.43]) compared to patients in the steroids-alone group (p = 0.01). While preserving graft viability in HR-PKP patients remains challenging, adjunctive topical tacrolimus combined with steroids demonstrates a clinically meaningful reduction in graft failure risk. Larger long-term studies are warranted to validate these findings.

## Introduction

Rejection prophylaxis following keratoplasty is crucial to ensuring the best possible long-term clinical outcomes. Although immunological rejection occurs in 10–30% of uncomplicated penetrating keratoplasty (PKP) cases, it remains a major challenge, with a significantly higher prevalence in high-risk PKP cases [1–4]. In fact, up to 60% of high-risk cases experience graft rejection episodes, and irreversible loss occurs in up to 70% of these cases within 10 years, despite prophylactic immunosuppressive therapy [5–7].

Steroid therapy remains the primary approach for preventing and managing corneal graft rejection [8]. While effective, with reported graft rejection episode reversal rates ranging from 40 to 90%, prolonged steroid use can lead to various ocular side effects, including elevated intraocular pressure, cataract and bacterial keratitis [7, 9, 10]. Moreover, steroids alone may not be the most optimal management option in high-risk PKP cases [9].

The Collaborative Corneal Transplantation Studies Research Group defined high-risk criteria as follows: a history of previous keratoplasty, stromal neovascularisation affecting two or more corneal quadrants, and/or active inflammation (evidenced by conjunctival/limbal redness with or without chemosis, ocular pain, discharge, or eyelid swelling) or perforation (whether infectious or non-infectious) present at the time of surgery [11–13].

In those high-risk PKP patients, calcineurin inhibitors, such as cyclosporine A and tacrolimus, have emerged as adjunctive or alternative immunosuppressive agents [14]. Compared to topical cyclosporine and steroids, topical tacrolimus has shown particularly promising results in preventing graft rejection, with a better safety profile [15, 16]. Specifically, topical tacrolimus has demonstrated higher efficacy in rejection prevention while inducing fewer adverse effects [6, 9, 10].

Therefore, our systematic review and meta-analysis aimed to summarise the literature on, and evaluate the efficacy of, adjunctive topical tacrolimus in reducing the risk of graft failure among high-risk PKP patients.

## Materials and methods

### Literature search

A systematic review and meta-analysis, registered with PROSPERO (ID: CRD42024585962), were conducted following the Preferred Reporting Items for Systematic Reviews and Meta-Analyses (PRISMA) guidelines [17]. PubMed, Web of Science and the Cochrane Central Register of Controlled Trials (CENTRAL), including Embase and ClinicalTrials.gov databases, were searched from inception to September 2024. A search using medical subject headings (MeSH) terms and keywords was conducted in each database, employing the Boolean operators ‘OR’ and ‘AND.’ The terms used included: (‘keratoplasty’ OR ‘corneal transplant’) AND (‘tacrolimus’ OR ‘FK506’ OR ‘cyclosporine’ OR ‘corticosteroids’) AND (‘randomised clinical trials’ OR ‘controlled trials’ OR ‘retrospective study’ OR ‘prospective study’ OR ‘case series’) (Supplementary Table 1).

### Study selection

Pre-established inclusion and exclusion criteria were deductively defined. Studies were included if they met the following criteria: (1) they were comparative randomised clinical trials or retrospective or prospective studies of patients who underwent high-rejection-risk penetrating keratoplasty (defined as having significant infectious diseases [bacterial, fungal, viral], inflammatory diseases, re-transplantations, or corneal neovascularisation due to chemical injury or previous infections); (2) they enrolled adults (≥18 years); and (3) they compared topical tacrolimus plus steroids (intervention) to either artificial tears plus topical steroids or topical steroids alone (comparator).

Studies were excluded if they included: (1) patients with normal-risk keratoplasty; (2) patients who underwent deep anterior lamellar keratoplasty or endothelial keratoplasty; (3) patients receiving systemic (intravenous or oral) tacrolimus or steroids as an intervention or comparator; and (4) fewer than five cases. Studies were also excluded if they were animal studies, case reports, meta-analyses, reviews, editorials, letters, or books; if they contained insufficient clinical data (i.e. lacking any of the following: patient demographics, management details, or outcomes); or if they were not written in English.

Two authors (JQ and RB) independently assessed the titles and abstracts of all extracted papers according to the inclusion and exclusion criteria. Studies that met inclusion criteria were then further evaluated through full-text review by the same two authors. Disagreements were resolved by a third author (SA).

### Data extraction and synthesis

Data from the included studies were extracted by two authors (RB and JQ) and independently verified by a third author (SA) to ensure accuracy. The extraction variables included: (1) author’s name, (2) date of publication, (3) level of evidence, (4) sample size, (5) age and sex, (6) clinical features, (7) type of intervention with doses and frequency, (8) type of comparator with doses and frequency, and (9) graft failure events. The primary outcome of interest was the incidence of graft failure, defined as the occurrence of irreversible rejection or other causes leading to transplant failure, as reported in the included studies.

### Statistical Analysis

Stata (StataCorp. 2025. *Stata Statistical Software: Release 19*. College Station, TX: StataCorp LLC) was used for all statistical analyses. Continuous variables were summarised as means and standard deviations (SD) or weighted means (effect size of means) and 95% confidence intervals (CIs), whereas categorical variables were summarised as frequencies and percentages or weighted proportions (effect size of proportions) and 95% CIs. The χ^2^ (z) test was used to assess significance among simple proportions. Weighted means, proportions and log odds ratios meta-analyses were conducted for pooled means, proportions and log odds ratios, using the random-effects model [18–21]. Subgroup analysis was performed to test statistical differences between groups. A leave-one-out sensitivity analysis of the odds-ratio meta-analysis was conducted to assess the robustness of the findings and evaluate whether any single included study unduly influenced the final results. A two-tailed p-value of <0.05 was considered statistically significant. The χ^2^ (z) test and the Higgins I^2^ test were used to assess heterogeneity among studies [22]. Funnel plots and Egger’s test (p < 0.05 indicating the presence of bias) were used to detect publication bias [23]. No publication bias was detected in any of the included studies (Supplementary Fig. 1).

## Results

### Study selection

**Fig. 1:**
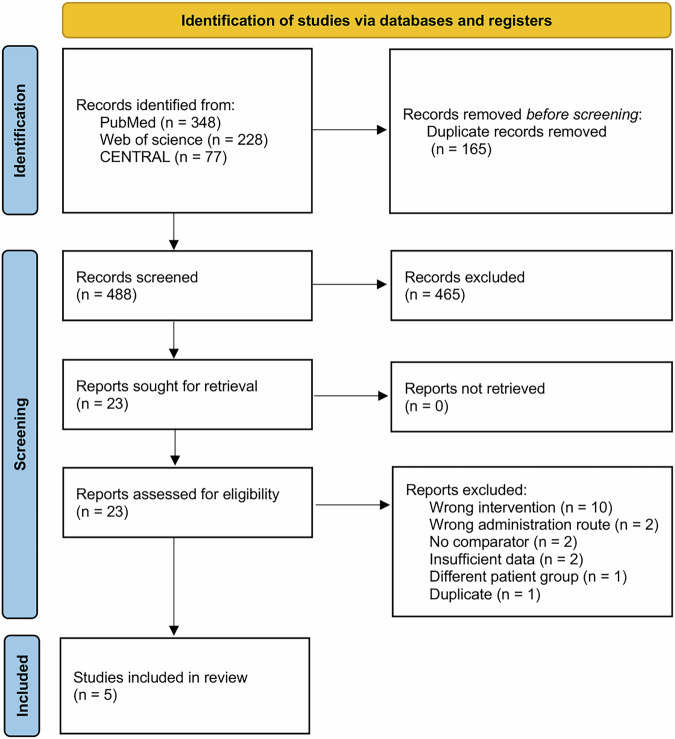
PRISMA flowchart. This figure illustrates the search strategy and data selection based on the inclusion and exclusion criteria.

Our initial literature search of databases yielded a total of 653 articles (Fig. 1). After removing duplicate records, 488 articles remained for title and abstract screening. Of these, 465 studies were subsequently excluded. Twenty-three papers were selected for full-text retrieval and assessed for inclusion, resulting in the exclusion of 18 articles that did not meet the inclusion criteria. The references of the retrieved articles were also reviewed during the full-text screening process to identify additional relevant studies, such as Rawat et al., which was not found on PubMed but was identified through manual screening of references^24^. Therefore, five articles were ultimately included in the analysis (Supplementary Table 2) [6, 9, 10, 24, 25].

The level of evidence for each article was assessed according to the 2011 Oxford Centre for Evidence-Based Medicine guidelines [26]. Hashemian et al. and Shimazaki et al. were categorised as level I evidence; Rawat et al. was categorised as level II evidence; and Bernardes et al. and Magalhaes et al. were categorised as level III evidence [6, 9, 10, 24, 25]. Risk of bias was assessed using the JBI tool (Supplementary Table 3) [27, 28].

### Demographics and clinical characteristics

We included a total of five homogeneous comparative studies, with a total of 274 patients who underwent high-risk penetrating keratoplasty (PKP). The tacrolimus-plus-steroids group comprised 138 patients, while the steroids-alone group included 136 patients (Table 1).

**Table 1:** Demographic and clinical characteristics of the cohort (n = 274).

Variable	Topical Tacrolimus plus Steroids (n = 138)	Topical Steroids (n = 136)	*p*-value
Age, years	54 (35.8–72.3)	52.6 (38.8–66.5)	0.90
Sex			
Male	60.1% (51.4–68.1%)	57.0% (44.7–68.5%)	0.68
Female	39.9% (31.9–48.6%)	43.0% (31.5–55.3%)	0.68
Underlying diagnosis			
Keratoconus	29.7% (16.6–47.2%)	25.6% (15.6–39.2%)	0.68
Scar	24.1% (7.4–55.7%)	29.0% (8.7–63.5%)	0.80
Microbial keratitis	23.0% (7.9–50.7%)	27.1% (19.3–36.7%)	0.74
Bullous keratopathy	19.2% (5.9–47.5%)	25.0% (17.1–35.1%)	0.64
Trauma	9.2% (4.7–17.0%)	11.2% (5.6–21.2%)	0.66
Chemical burn	8.9% (4.5–16.9%)	7.1% (3.2–15.0%)	0.66
Follow-up duration, months^a^	20.1 (11.7–28.5)	20.2 (10.2–30.2)	0.98

All data are reported as weighted means (95% confidence intervals) or logit-proportions % (95% confidence intervals), derived from meta-analyses.

^a^The mean follow-up duration was reported only by Hashemian et al. [10] and Magalhaes et al. [25], whereas the other three studies reported either the total or median follow-up period, as detailed in Supplementary Table 1.

There were no statistically significant differences in the demographic data between the two groups (Table 1). The weighted mean age for the tacrolimus-plus-steroids group was 54 years (95% CI: 35.8–72.3), compared to 52.6 years in the steroids-alone group (95% CI: 38.8–66.5, p = 0.90). There was an overall male predominance in both groups (tacrolimus-plus-steroids group: 60.1% [95% CI: 51.4–68.1%]; steroids-alone group: 57.0% [95% CI: 44.7–68.5%], p = 0.68; Table 1).

The underlying diagnoses were similar in the two groups. Keratoconus was the most common diagnosis, present in 29.7% (95% CI: 16.6–47.2%) of the tacrolimus-plus-steroids group and 25.6% (95% CI: 15.6–39.2%) of the steroids-alone group (p = 0.68; Table 1). Other diagnoses, including scar (24.1% vs. 29.0%, p = 0.80), microbial keratitis (23.0% vs. 27.1%, p = 0.74), bullous keratopathy (19.2% vs. 25.0%, p = 0.64), trauma (9.2% vs. 11.2%, p = 0.66) and chemical burn (8.9% vs. 7.1%, p = 0.66).

The weighted mean follow-up duration was comparable between the tacrolimus-plus-steroids group (20.1 months [95% CI: 11.7–28.5]) and the steroids-alone group (20.2 months [95% CI: 10.2–30.2], p = 0.98; Table 1).

### Topical tacrolimus: graft outcome in high-risk penetrating keratoplasty

**Fig. 2:**
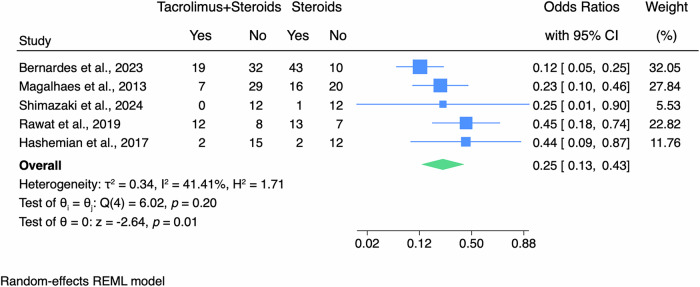
Forest plots of graft failure outcomes. CI, confidence interval; REML, restricted maximum likelihood.

Pooling odds ratios (OR) of graft failure for both the tacrolimus-plus-steroids group and the steroids-alone group resulted in an overall statistically significant finding. After undergoing high-risk PKP, patients in the tacrolimus-plus-steroids group had significantly lower odds of graft failure by 75.0% (OR: 0.25 [95% CI: 0.13–0.43]) compared to patients in the steroids-alone group (p = 0.01; Fig. 2).

**Fig. 3:**
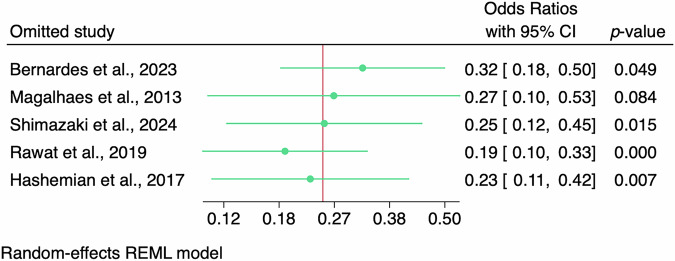
Leave-one-out sensitivity analysis for the odds-ratio meta-analysis of graft failure. This figure shows the outcomes comparing topical tacrolimus plus steroids vs. topical steroids alone. CI, confidence interval; REML, restricted maximum likelihood.

Leave-one-out sensitivity analysis showed that the pooled effect estimate (OR) remained statistically significant and directionally consistent regardless of which study was excluded (OR range: 0.19–0.32), supporting the robustness of the findings (Fig. 3). When Magalhaes et al. [25] was omitted, the pooled OR increased slightly (OR: 0.27 [95% CI: 0.10–0.53], p = 0.084). While this represents a borderline loss of statistical significance, the CI remained below the null (OR = 1) and the effect direction was consistent with the overall finding (Fig. 3).

## Discussion

To the best of our knowledge, this meta-analysis represents the first systematic comparison of adjunctive topical tacrolimus combined with topical steroids versus steroids alone for postoperative management in high-risk PKP patients. The tacrolimus-plus-steroids (n = 138) and steroids-alone (n = 136) groups were well-matched in baseline characteristics, including weighted mean age (p = 0.90), sex distribution (p = 0.68) and underlying indications for PKP, with keratoconus being the most common (p = 0.68). Follow-up durations were also similar between groups (20.1 vs. 20.2 months, p = 0.98), reducing the likelihood of demographic or clinical confounding factors potentially influencing the results. Our findings show that the addition of topical tacrolimus to steroid therapy may improve corneal graft survival in these high-risk patients.

### Tacrolimus: immunosuppressive profile

Used in the prophylaxis and treatment of post-transplant rejection, tacrolimus is an immunosuppressive agent that mainly works by inhibiting calcineurin [29]. It selectively hinders T-cell proliferation by binding to FK506 binding protein, leading to the cessation of the body’s immunological rejection of a new transplant [29, 30]. Due to its efficacy, tacrolimus has been used alone or in combination with other immunosuppressive agents to reduce the likelihood of allograft rejections in several solid organ transplants, including heart, lung, liver and kidney transplants [31–34]. Moreover, tacrolimus has additional off-label uses in some dermatological and gastrointestinal autoimmune diseases [35].

While known for its efficacy in organ transplants, systemic tacrolimus may cause alarming adverse effects, including nephrotoxicity, headache, abdominal pain and blurred vision, depending on the route of administration, dosage and frequency [30]. Furthermore, as it has a narrow therapeutic index, continuous and close monitoring is mandatory for transplant patients, primarily to minimise drug-mediated nephrotoxicity and other adverse effects [36–38]. Interdisciplinary management is generally recommended for post-transplant patients receiving systemic tacrolimus, particularly those at high risk of rejection [39, 40].

### Topical tacrolimus: efficacy in immune-mediated reactions and transplant survival

Recently, the prophylactic and therapeutic use of tacrolimus in ophthalmic conditions has emerged. Compared to other immunosuppressive agents, such as topical cyclosporine A, topical tacrolimus has demonstrated ten to a hundredfold better efficacy in managing immune-mediated anterior segment disorders [41]. For example, topical tacrolimus effectively controlled intraocular inflammation in patients with both infectious and noninfectious uveitis [42, 43]. Furthermore, a meta-analysis of randomised clinical trials conducted by Zhao et al. found that topical tacrolimus significantly reduced ocular objective signs and subjective symptom evaluation scores in patients with vernal keratoconjunctivitis [44]. Other uses, such as allergic eye disease, scleritis, chronic ocular graft-versus-host disease and ocular cicatricial pemphigoid, have also been reported to improve patients’ clinical outcomes [42].

While no universally standardised guidelines exist for steroid and tacrolimus use in high-risk PKP patients, several studies reported their clinical experience. In a 2007 case series by Mason et al., four patients with high-risk PKP received topical tacrolimus 0.03% ointment, which was well-tolerated and effective in all cases [45]. More recently, Qi et al. implemented a structured tapering protocol using 0.1% tacrolimus eye drops four times daily for the first month, followed by gradual tapering to three times daily for 6 months, then twice daily for at least 1 year [16]. These regimens are similar to those reported by the included studies in our review (Supplementary Table 2); however, further long-term studies and standardised protocols are needed to guide the topical steroid and tacrolimus management in high-risk corneal transplants.

A retrospective cohort study of 14 children receiving systemic, followed by topical, tacrolimus after undergoing PKP reported a higher 1-year graft survival rate (37.5%) compared to previously published literature [46]. A similar comparative study led by Tran et al. in patients undergoing primary keratolimbal transplantation for limbal stem cell deficiency found that tacrolimus showed favourable post-keratoplasty graft failure rates regardless of the administration route (oral tacrolimus: 9.1 per 1000 person-months versus topical tacrolimus: 8.4 per 1000 person-months, p = 0.96]) [47]. Similarly, in patients undergoing high-risk PKP, our meta-analysis demonstrated the potential efficacy of adjunctive topical tacrolimus to standard steroid therapy in reducing the risk of corneal transplant failure. Furthermore, we found that patients receiving the topical tacrolimus-plus-steroid regimen post-PKP were 75.0% less likely to experience graft failure.

In our meta-analysis, compared to receiving standard steroids alone after high-risk PKP, patients receiving both topical tacrolimus and steroids demonstrated significantly lower odds of graft failure (OR: 0.25 [95% CI: 0.13–0.43], p = 0.01). These findings are consistent with those of Qi et al., who concluded that topical tacrolimus showed a significantly higher 3-year post-keratoplasty survival rate (81.6% ± 5.3%) compared to topical cyclosporine (71.1% ± 6.3%, p = 0.006) [16]. Similarly, a randomised clinical trial conducted by Zhai et al. reported that topical tacrolimus increased corneal graft survival with fewer adverse effects compared to topical cyclosporine [15]. These findings highlight the clinical efficacy of incorporating adjunctive topical tacrolimus with standard steroid therapy in the postoperative management of high-risk corneal transplant recipients to maintain their graft viability and mitigate failure risk.

### Limitations

This meta-analysis was limited by selection biases. The small sample sizes in some studies could reduce statistical power for multiple endpoints. Additionally, variations in tacrolimus and steroids dosing regimens across studies may limit the generalisability of our conclusions. Despite these limitations, our study has notable strengths, including the inclusion of homogeneous comparative studies, the use of a random-effects model to account for variability and sensitivity analyses confirming the robustness of our results. Further comprehensive, long-term interventional studies are warranted to: (1) establish the optimal topical tacrolimus-plus-steroids regimen for high-risk PKP and (2) investigate factors affecting graft survival and quality of life in this group of patients.

## Conclusion

While high-rejection-risk patients often face challenges in preserving their corneal transplant, the addition of topical tacrolimus to standard steroid therapy may significantly reduce the risk of graft failure after PKP. Our findings support the use of topical tacrolimus as a standard adjunctive therapy for high-risk transplant patients to improve graft survival and overall clinical outcomes. Although we systematically summarised the available evidence, the relatively small sample sizes of the included studies may limit the generalisability of our results. Therefore, further large, long-term studies to evaluate the efficacy of adjunctive topical tacrolimus with steroids in high-risk PKP are needed to draw more conclusive recommendations.

## Supplementary information

Supplementary Tables 1–3

Supplementary Figure 1. Funnel plot of graft failure outcomes. CI, confidence interval.

## Data Availability

The datasets generated during and/or analysed during the current study are available from the corresponding author on reasonable request.
