# Supplementary material for: Efficacy of Topical Tacrolimus (FK506) in High-risk Penetrating Keratoplasty: A Systematic Review and Meta-analysis of Comparative Studies
Source: Eye (Lond). 2025 Oct 26;39(18):3237–42. doi: 10.1038/s41433-025-04002-x (PMC12669711; doi:10.1038/s41433-025-04002-x)
Supplement: Supplementary file 1 — Supplementary Tables 1–3 [file 41433_2025_4002_MOESM1_ESM.docx]

**Supplementary Table 1.** Search strategy for PubMed, Web of Science, and Cochrane library databases

**PubMed (**Date run: 1 September 2024)

| #1 | keratoplasty | 25,730 |
| --- | --- | --- |
| #2 | corneal transplant | 23,051 |
| #3 | (keratoplasty) OR (corneal transplant) | 26,229 |
| #4 | tacrolimus | 29,447 |
| #5 | FK506 | 31,379 |
| #6 | cyclosporine | 63,135 |
| #7 | corticosteroids | 392,601 |
| #8 | (((corticosteroids) OR (cyclosporine)) OR (FK506)) OR (Tacrolimus) | 469,132 |
| #9 | randomized clinical trials | 811,153 |
| #10 | controlled trials | 1,091,875 |
| #11 | retrospective | 1,560,535 |
| #12 | prospective | 1,122,360 |
| #13 | case series | 185,010 |
| #14 | ((((case series) OR (prospective)) OR (retrospective)) OR (controlled trials)) OR (randomized clinical trials) | 3,569,838 |
| #15 | ((((((case series) OR (prospective)) OR (retrospective)) OR (controlled trials)) OR (randomized clinical trials)) AND ((((corticosteroids) OR (cyclosporine)) OR (FK506)) OR (Tacrolimus))) AND ((corneal transplant) OR (keratoplasty)) | 348 |

**Web of Science (**Date run: 1 September 2024)

| #1 | (ALL=(Keratoplasty)) OR ALL=(Corneal transplant) | 18,520 |
| --- | --- | --- |
| #2 | (((ALL=(tacrolimus)) OR ALL=(FK506)) OR ALL=(corticosteroids)) OR ALL=(cyclosporine) | 228,700 |
| #3 | ((((ALL=(randomized clinical trials)) OR ALL=(controlled trials)) OR ALL=(Retrospective )) OR ALL=(Prospective )) OR ALL=(case series) | 3,004,782 |
| #4 | #1 AND #2 AND #3 | 228 |

**Cochrane Central Register of Controlled Trials (CENTRAL) (**Date run: 1 September 2024)

| #1 | keratoplasty | 796 |
| --- | --- | --- |
| #2 | corneal transplant | 191 |
| #3 | #1 OR #2 | 899 |
| #4 | tacrolimus | 6049 |
| #5 | FK506 | 352 |
| #6 | corticosteroids | 17779 |
| #7 | cyclosporine | 6583 |
| #8 | #4 OR #5 OR #6 OR #7 | 27780 |
| #9 | randomized clinical trials | 971711 |
| #10 | controlled trials | 2202560 |
| #11 | retrospective | 44428 |
| #12 | prospective | 293189 |
| #13 | case series | 8686 |
| #14 | #9 OR #10 OR #11 OR #12 OR #13 | 2202857 |
| #15 | #3 AND #8 AND #14 | 77 |

| **Supplementary Table 2.** Characteristics of included studies | | | | | | | | | | | | |
| --- | --- | --- | --- | --- | --- | --- | --- | --- | --- | --- | --- | --- |
| **Study** | **Country** | **Design** | **No. of patients** | **Standard Therapy^*^** | **Intervention Group** | | | | **Comparator Group** | | | |
|  |  |  |  |  | **Male**  **(%)** | **Mean age (SD)** | **Intervention** | **F/U in months** | **Male**  **(%)** | **Mean age (SD)** | **Comparator** | **F/U in months** |
| Bernardes et al., 2023^1^ | Portugal | R | 106 | Oral methylprednisolone (tapered in 2-3 weeks) | 28 (52.8) | N/R | Topical Tacrolimus (0.2 mg/g); BID | Median: 30 (range: 12-43.5) | 25 (47.2) | N/R | Topical Dexamethasone (0.1 mg/g); 6ID | Median: 24 (range: 11-54) |
| Magalhaes et al., 2013^2^ | Brazil | R | 72 | Oral prednisone | 23 (63.9) | 52.7 (20.9) | Topical Tacrolimus (0.03%); BID | Mean: 23.1 (SD: 8.4) | 23 (63.9) | 48.3 (15.3) | Topical Prednisolone (1.0%); 8ID | Mean: 24 (SD: 12.4) |
| Rawat et al., 2019^3^ | India | P | 40 | Topical prednisolone acetate | 13 (65.0) | 53.5 (16.4) | Topical Tacrolimus (0.03%); BID | Total: 6 | 9 (45.0) | 49.45 (15.3) | Topical Prednisone (1.0%); q2h | Total: 6 |
| Hashemian et al., 2018^4^ | Iran | RCT | 31 | Topical prednisolone acetate and oral prednisolone | 10 (58.8) | 46.3 (20.5) | Topical Tacrolimus (0.05%); 4ID | Mean: 19 (SD: 5) | 13 (92.9) | 36.6 (15.7) | Artificial Tears (+ topical steroids as standard therapy) | Mean: 19.4 (SD: 5.6) |
| Shimazaki et al., 2024^5^ | Japan | RCT | 25 | Topical betamethasone phosphate and fluorometholone drops | 11 (91.7) | 61.4 (17.7) | Topical Tacrolimus (0.1%); BID | Total: 13 | 8 (61.5) | 65.6 (11.6) | Artificial Tears (+ topical steroids as standard therapy) | Total: 13 |
| RCT, randomized clinical trial; R, retrospective study; P, prospective study; SD, standard deviation; N/R, not reported; F/U, follow-up period.  ^*^All patients in both groups received standard therapy, classifying them as either topical tacrolimus plus steroids or topical steroids alone. | | | | | | | | | | | | |

**Supplementary Table 3.** Risk of bias assessments for included studies

| **Cohort studies JBI bias assessment tool** | **Bernardes et al., 2023^1^** | **Magalhaes et al., 2013^2^** | **Rawat et al., 2019^3^** |
| --- | --- | --- | --- |
| Were the two groups similar and recruited from the same population? | Yes | Yes | Yes |
| Were the exposure measured similarly to assign people to both exposed and unexposed groups? | Yes | Yes | Yes |
| Was the exposure measured in a valid and reliable way? | Yes | Yes | Yes |
| Were confounding factors identified? | No | No | No |
| Were strategies to deal with confounding factors stated? | N/A | N/A | N/A |
| Were the groups/participants free of the outcome at the start of the study (or at the moment of exposure)? | Yes | Yes | Yes |
| Were the outcomes measured in a valid and reliable way? | Yes | Yes | Yes |
| Was the follow up time reported and sufficient to be long enough for outcomes to occur? | Yes | Yes | Yes |
| Was follow up complete, and if not, were the reasons to loss to follow up described or explored? | Yes | Yes | Yes |
| Were strategies to address incomplete follow up utilized? | Yes | N/A | Yes |
| Was appropriate statistical analysis used? | Yes | Yes | Yes |
| Overall Appraisal | Include | Include | Include |

| **Randomized controlled trials JBI bias assessment tool** | **Hashemian et al., 2017^4^** | **Shimazaki et al., 2024^5^** |
| --- | --- | --- |
| Was true randomization used for assignment of participants to treatment groups? | Yes | Yes |
| Was allocation to treatment groups concealed? | Yes | Yes |
| Were treatment groups similar at the baseline? | Yes | Yes |
| Were participants blind to treatment assignment? | Yes | Yes |
| Were those delivering treatment blind to treatment assignment? | Yes | No |
| Were outcomes assessors blind to treatment assignment? | Yes | Yes |
| Were treatment groups treated identically other than the intervention of interest? | Yes | Yes |
| Was follow up complete and if not, were differences between groups in terms of their follow up adequately described and analyzed? | Yes | Yes |
| Were participants analyzed in the groups to which they were randomized? | Yes | Yes |
| Were outcomes measured in the same way for treatment groups? | Yes | Yes |
| Were outcomes measured in a reliable way? | Yes | Yes |
| Was appropriate statistical analysis used? | Yes | Yes |
| Was the trial design appropriate, and any deviations from the standard RCT design (individual randomization, parallel groups) accounted for in the conduct and analysis of the trial? | Yes | Yes |
| Overall Appraisal | Include | Include |

**References**

1. Bernardes L, Gil J, Costa E, et al. Topical tacrolimus in high-risk corneal transplants. *Eur J Ophthalmol*. 2024;34(1):140-145. doi:10.1177/11206721231172236
2. Magalhaes OA, Marinho DR, Kwitko S. Topical 0.03% tacrolimus preventing rejection in high-risk corneal transplantation: a cohort study. *Br J Ophthalmol*. 2013;97(11):1395-1398. doi:10.1136/BJOPHTHALMOL-2013-303639
3. Rawat DP. Comparative Study of Topical Tacrolimus with Topical Cyclosporine Therapy on Graft Survival and Visual Outcome in Penetrating keratoplasty. *journal of Medical Science And clinical Research*. 2019;7(4). doi:10.18535/JMSCR/V7I4.16
4. Hashemian MN, Latifi G, Ghaffari R, et al. Topical Tacrolimus as Adjuvant Therapy to Corticosteroids in Acute Endothelial Graft Rejection After Penetrating Keratoplasty: A Randomized Controlled Trial. *Cornea*. 2018;37(3):307-312. doi:10.1097/ICO.0000000000001408
5. Shimazaki J, Tomida D, Yagi-Yaguchi Y, Satake Y, Yamaguchi T. Topical tacrolimus for high-risk corneal transplantation: a randomized, clinical trial. *BMC Ophthalmol*. 2024;24(1). doi:10.1186/S12886-024-03506-6
