# Supplementary figures and images for: Efficacy of Topical Tacrolimus (FK506) in High-risk Penetrating Keratoplasty: A Systematic Review and Meta-analysis of Comparative Studies
Source: Eye (Lond). 2025 Oct 26;39(18):3237–42. doi: 10.1038/s41433-025-04002-x (PMC12669711; doi:10.1038/s41433-025-04002-x)

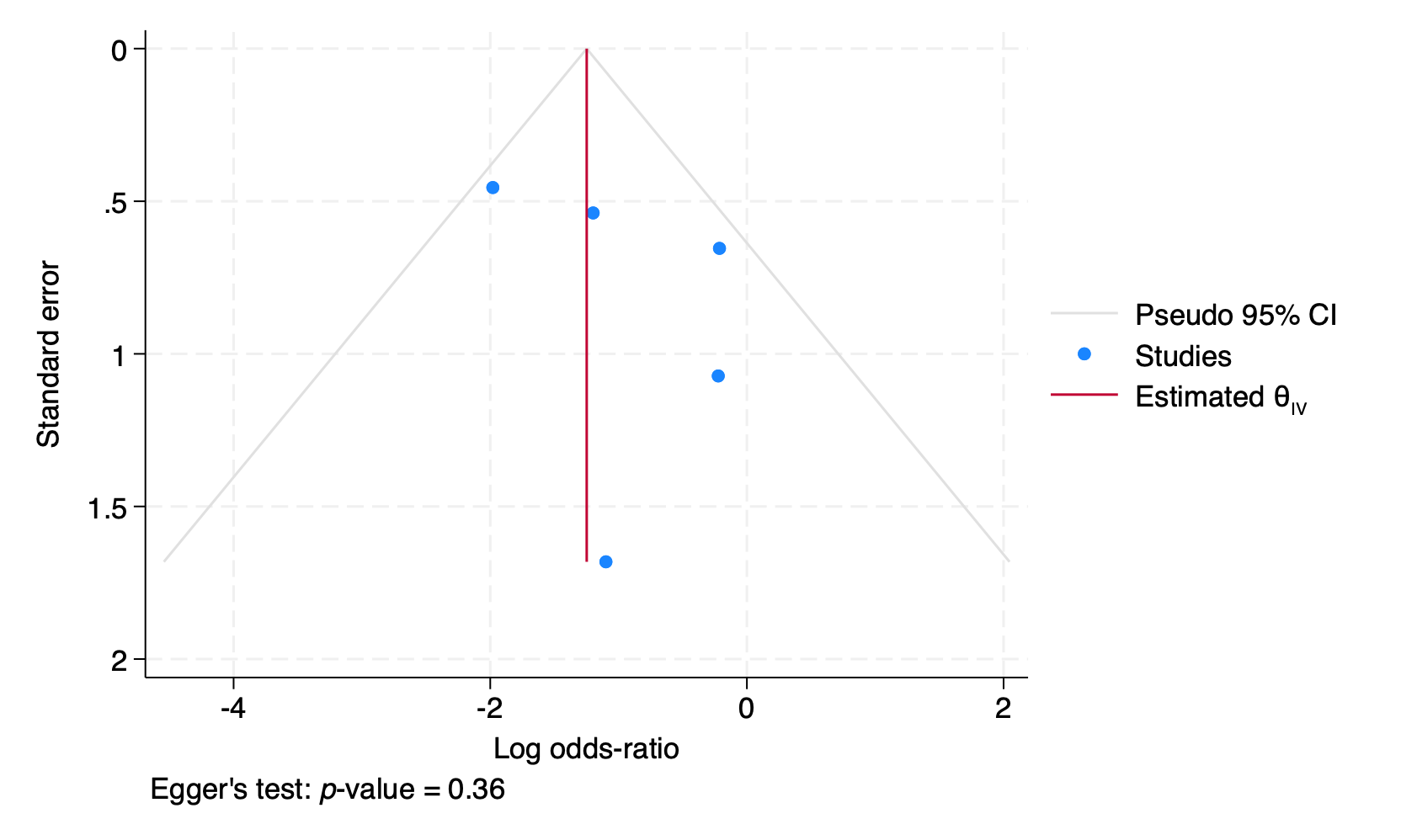

Supplement: Supplementary file 2 — Supplementary Figure 1. Funnel plot of graft failure outcomes. CI, confidence interval. [file 41433_2025_4002_MOESM2_ESM.tif]
